# Regulation of Autophagy by Nuclear GAPDH and Its Aggregates in Cancer and Neurodegenerative Disorders

**DOI:** 10.3390/ijms20092062

**Published:** 2019-04-26

**Authors:** Giovanna Butera, Nidula Mullappilly, Francesca Masetto, Marta Palmieri, Maria Teresa Scupoli, Raffaella Pacchiana, Massimo Donadelli

**Affiliations:** 1 Department of Neurosciences, Biomedicine and Movement Sciences, Section of Biochemistry, University of Verona, Strada Le Grazie 8, 37134 Verona, Italy; giovanna.butera@univr.it (G.B.); nidula.mullappilly@univr.it (N.M.); francesca.masetto@univr.it (F.M.); marta.palmieri@univr.it (M.P.); mariateresa.scupoli@univr.it (M.T.S.); 2 Research Center LURM (Interdepartmental Laboratory of Medical Research), University of Verona, 37134 Verona, Italy

**Keywords:** GAPDH, autophagy, cancer, aggregates, cell death

## Abstract

Several studies indicate that the cytosolic enzyme glyceraldehyde-3-phosphate dehydrogenase (GAPDH) has pleiotropic functions independent of its canonical role in glycolysis. The GAPDH functional diversity is mainly due to post-translational modifications in different amino acid residues or due to protein–protein interactions altering its localization from cytosol to nucleus, mitochondria or extracellular microenvironment. Non-glycolytic functions of GAPDH include the regulation of cell death, autophagy, DNA repair and RNA export, and they are observed in physiological and pathological conditions as cancer and neurodegenerative disorders. In disease, the knowledge of the mechanisms regarding GAPDH-mediated cell death is becoming fundamental for the identification of novel therapies. Here, we elucidate the correlation between autophagy and GAPDH in cancer, describing the molecular mechanisms involved and its impact in cancer development. Since autophagy is a degradative pathway associated with the regulation of cell death, we discuss recent evidence supporting GAPDH as a therapeutic target for autophagy regulation in cancer therapy. Furthermore, we summarize the molecular mechanisms and the cellular effects of GAPDH aggregates, which are correlated with mitochondrial malfunctions and can be considered a potential therapeutic target for various diseases, including cancer and neurodegenerative disorders.

## 1. Introduction

Autophagy is a physiological degradative mechanism of the cells by which autophagic vesicles deliver unfolded proteins and damaged organelles to lysosomes for their elimination. In this way, autophagy induces the degradation and recycling of cellular components to support energy metabolism in stressing conditions and cellular homeostasis [1]. Accumulating evidence also demonstrates that autophagy declines with age and that impaired autophagy favors individuals to age-related diseases, whereas interventions that stimulate autophagy can promote longevity [2]. However, the excessive stimulation of autophagy can be considered a self-eating mechanism favoring cell death and the aberrant regulation of autophagy is widely studied to improve therapeutic treatments for various diseases including cancer [3]. In different stages of tumorigenesis, autophagy may have opposite and context-dependent roles acting as tumor suppressor or cancer promoter [4]. Many external stimuli may affect autophagy in cancer, such as hypoxia, acidification of tumor microenvironment, nutrient deprivation, drug therapies, or infections [5,6]. A global analysis of the mutational status of the genes encoding the entire core autophagy machinery reveals that it is generally not targeted by high-frequency somatic single-nucleotide mutations across cancers [7]. These large-scale analyses indicate that the core autophagy machinery largely escapes genomic mutations and cancer cells generally express a functionally intact autophagy pathway. An exception to this general scheme is represented by the tumor suppressor BECN1 (ATG6), which lost with high frequency one allele in various cancer types [8]. However, BECN1 is adjacent to the known tumor suppressor gene breast cancer 1 (BRCA1) on chromosome 17. Hereditary breast cancer commonly results from the presence of a pathogenic germline missense mutation in BRCA1 followed by somatic deletion of the remaining wild-type BRCA1 allele. These deletions are typically large, deleting BRCA1 along with hundreds of other genes, including BECN1. Further studies demonstrated that BRCA1 loss is the driver mutation in hereditary and sporadic breast cancer [9], casting doubt on BECN1′s role as a tumor suppressor. In cancers, the autophagic flux can be modulated by tumor suppressors or oncogenes. A crucial example is that of p53, the status of which can modify the role of autophagy in tumor progression [10,11]. Furthermore, several studies indicate that p53 triggers autophagy in cancer cells through various mechanisms, such as the stimulation of AMPK, the inhibition of mTOR, or the induction of the autophagy-related gene DRAM1, to react to genotoxic or environmental stimuli [12,13]. On the other side, mutant p53 proteins counteract the autophagic machinery by various molecular mechanisms, including the transcriptional repression of ATG12 [14]. Notably, mutant p53 protein stability is affected by lysosome-mediated degradation through autophagy, supporting the concept of a functional crosstalk between mutant p53 proteins and autophagy in cancer progression [15].

Curiously, even a glycolytic enzyme, i.e., glyceraldehyde-3-phosphate dehydrogenase (GAPDH), may regulate autophagy and cell death through mechanisms that we summarize in this review. In addition to the main glycolytic role of the tetrameric conformation of GAPDH, which catalyzes the reversible conversion of glyceraldehyde-3-phosphate to 1,3-bisphosphoglycerate, GAPDH serves as a versatile enzyme that plays several regulatory roles determining the fate of the cells [16]. Indeed, GAPDH performs pleiotropic functions that mainly depend on its subcellular localization [17]. In addition to the cytosolic distribution of the glycolytic enzyme, GAPDH can also be detected close to the plasma membrane, mitochondria, nucleus, endoplasmic reticulum (ER), polysomes, Golgi and even secreted in the extracellular space carrying out several key functions including the regulation of mRNA stability [18], iron uptake and transport [19], DNA repair [20], nuclear tRNA export [21], cell death [22] and intracellular membrane trafficking [23]. The GAPDH multifunctional properties can be regulated by several mechanisms, such as the formation of macromolecular complexes by protein–protein interactions; post-translational modifications, e.g., acetylation, phosphorylation and nitrosylation; or protein oligomerization. The oligomeric state of GAPDH and its propensity to aggregate is mainly dependent on various signal molecules [24]. The redox sensitive cysteine residues of the enzyme, which includes Cys-152 in the active site, are also target of reactive oxygen species (ROS) or reactive nitrogen species (RNS) and, consequently, GAPDH aggregation is influenced by several other stimuli inducing cellular oxidative/nitrosative stresses [24,25]. Besides cancer, the functional versatility of this enzyme determines that GAPDH alteration is involved in several other diseases [26] especially neurodegenerative disorders such as Alzheimer’s disease (AD), Parkinson’s disease (PD) and Huntington’s disease (HD) [27].

In this review, we summarize the role of GAPDH in regulating autophagy and cell death in cancer cells mainly through its nuclear translocation and the effects of its aggregation in several degenerative disorders. Finally, we propose that the modulation of the subcellular localization of GAPDH can be considered a potential target mechanism in autophagy regulation for cancer therapy.

## 2. Non-Glycolytic Roles of GAPDH

Due to the non-glycolytic roles of GAPDH, this enzyme is considered a moonlight protein in the cells. These non-glycolytic roles include physio-pathological functions such as regulation of gene expression, DNA repair and replication, neurodegeneration, pathogenesis, virulence in bacteria, tubular bundling, protein–protein interactions, RNA export, as well as apoptosis and autophagy. For instance, it has been discovered that GAPDH acts as a key component of the co-activator complex of Oct-1 in the transcriptional induction of histone H2B gene during the S phase of the cell cycle. Intriguingly, GAPDH interacts directly with Oct-1 and it has an intrinsic activation domain that can relate with the general transcription machinery [28]. This proof-of-concept opened the way to the investigation of target genes which can be transcriptionally regulated by nuclear GAPDH.

Concerning autophagy, it has been demonstrated that GAPDH may also act as a glucose sensor in the cells stimulating autophagic degradation. Indeed, during glucose starvation, the AMPK-dependent GAPDH phosphorylation is essential for SIRT1 activation and stimulation of autophagy. In these conditions, cytoplasmic GAPDH is phosphorylated by activated AMPK prompting GAPDH to redistribute into the nucleus. Inside the nucleus, GAPDH directly interacts with SIRT1, displacing SIRT1’s repressor and increasing SIRT1 deacetylase activity [29]. In general, the multiple activities of GAPDH are related to its translocation to the nucleus or to different subcellular compartments in addition to the cytosolic localization, where its main role in glycolysis is well characterized.

## 3. Subcellular Localization of GAPDH

**Figure 1:**
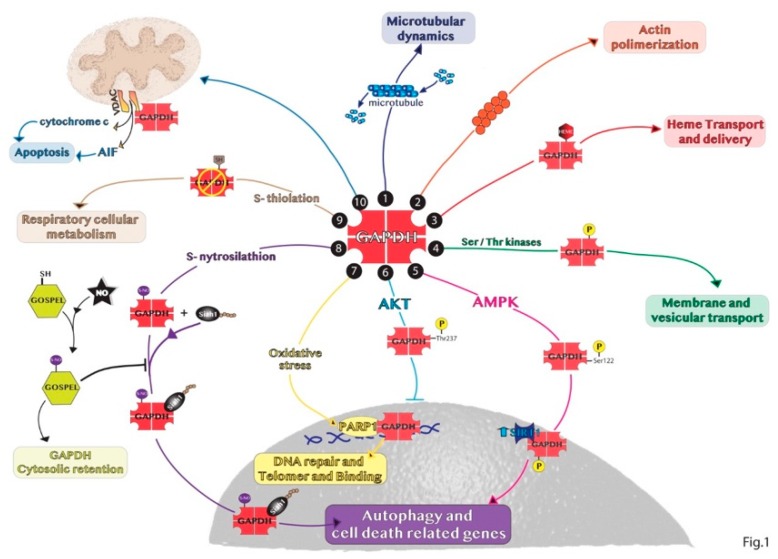
Representative summary of the non-glycolytic roles of GAPDH. (1) GAPDH interaction with microtubule helps in microtubular dynamics. (2) GAPDH favors actin polymerization. (3) GAPDH acts as chaperone with endogenous and exogenous heme. (4) GAPDH phosphorylation is involved in early secretory pathway transport. (5) In response to glucose deprivation, activated AMPK phosphorylates GAPDH in the cytosol inducing GAPDH translocation to the nucleus and its binding with SIRT1 promoting autophagy. (6) GAPDH phosphorylation by AKT blocks the translocation of the enzyme to the nucleus. (7) In the nucleus, GAPDH binds to PARP1 or damaged DNA to induce DNA repair. (8) SNO-GAPDH binds Siah1 and the complex translocates into the nucleus inducing cell death related genes. (9) GAPDH glycolytic activity can be inhibited by S-thiolation increasing respiratory cellular metabolism. (10) GAPDH binds VDAC1 channel favoring apoptosis by releasing cytochrome c and AIF.

The subcellular and glycolytic-independent redistribution of the enzyme can mainly occur in the nucleus, mitochondria, and intra- and extra-cellular vesicles. The regulation of GAPDH compartmentalization is mainly due to its oligomerization, post-transcriptional modifications and protein–protein interactions, thus defining also the non-glycolytic functions of GAPDH. These findings are schematically represented in Figure 1.

### 3.1. Nuclear Roles of GAPDH

Nuclear GAPDH is involved in a variety of functions such as autophagy and cell death, DNA repair, protection of telomeres from rapid degradation. The accumulation into the nucleus of GAPDH promotes the decline of its glycolytic activity [16]. During oxidative stress, when DNA is damaged, simultaneous nitrosylation and translocation of GAPDH to the nucleus takes place and it can either bind to poly(ADP-ribose) polymerase 1 (PARP1) or directly to the damaged DNA. Under these stress conditions, PARP1 is activated by damaged DNA and synthesizes poly(ADP-ribose) using NAD^+^. Moreover, GAPDH translocated to the nucleus binds and activates PARP1. Over activation of PARP1 depletes intracellular NAD^+^, therefore the NAD^+^ binding site of GAPDH becomes free and the enzyme acquires the ability to bind DNA. If a single stranded DNA fragment contains a cleaved site, GAPDH forms a stable covalent adduct with this damage [30]. Thus, the formation of an irreversible complex of GAPDH with DNA seems to be a suicidal event, which hampers DNA repair in the case of accumulation of several damages and can be a factor leading to cell death.

A study performed in human lung carcinoma indicates that the NAD^+^ binding domain of GAPDH is associated with telomere and that telomere binding activity of nuclear GAPDH is regulated by sphingolipid ceramide in a cell cycle dependent manner [31]. Moreover, a study in HEK293 early embryonic kidney cells suggests that nuclear translocation of the enzyme happens only during early apoptosis preceding the changes in chromatin, and not in late apoptosis phase [32]. The signal from the cellular stress is carried over by nitrosylated GAPDH and by its binding to the E3 ubiquitin ligase Siah1, favoring their nuclear translocation as a complex. Indeed, in response to oxidative stress, GAPDH becomes a target for *S*-nitrosylation (SNO), which abolishes its glycolytic activity modifying the conformation of the active site of the enzyme [33,34]. Binding SNO-GAPDH, Siah1 acquires a longer half-life and can be translocated to the nucleus [35]. The balance among GAPDH and its *S*-nitrosylated form is regulated by GOSPEL, which is *S*-nitrosylated before it thus preserving GAPDH glycolytic function and preventing the interaction with Siah1. However, above a nitrosative stress threshold, GOSPEL cannot avoid GAPDH/Siah1 complex formation leading to apoptosis [36]. Thus, GAPDH facilitates the stabilization of Siah1, causing the degradation of nuclear proteins and consequent cell death [35]. Nuclear translocation of GAPDH and consequent cell death are also facilitated by the acetylation of lysine residues 117 and 251 with the involvement of acetyl transferase PCAF located in the nucleus [37]. This further acetylates apoptotic targets, as tumor suppressor p53, PUMA, BAX and p21 [37].

In pancreatic cancer cells, our group found a strong increase in GAPDH nuclear translocation due to oxidation of the enzyme by increased ROS after combined treatment with genipin and everolimus drugs. Nuclear GAPDH positivity was functionally associated to cancer cell death in vitro and in vivo model of pancreatic cancer [38]. In this tumor type, we have also shown that oncogenic mutant p53 contributes to enhance the glycolytic pathway blocking the nuclear GAPDH translocation. This supports ATP production and the formation of metabolic intermediates for cancer cell growth. We also demonstrated that mutant p53 adopts different strategies to prevent nuclear translocation of the enzyme by forming the SIRT1/GAPDH complex, as well as by the regulation of AMPK and AKT signaling pathways, which in turn regulate GAPDH translocation in the nucleus by direct phosphorylation [39]. In the mouse embryonic fibroblasts, following the disruption of glucose intake, the GAPDH was found to be translocated from cytosol to nucleus followed by formation of LC3 positive puncta, a marker of autophagy [29]. Indeed, in response to glucose deprivation, activated AMPK phosphorylates GAPDH at Ser122 in the cytoplasm and the phosphorylated GAPDH moves into the nucleus and directly interacts and activates SIRT1, resulting in autophagy stimulation. The authors suggested that this nuclear GAPDH-driven mechanism may launch autophagy to supply energy or remove aberrant proteins or organelles, such as damaged mitochondria at an early stage of cellular stress. When the stress is sustained, more GAPDH moves into the nucleus to induce apoptosis and cell death.

Moreover, GAPDH has shown an intrinsic role in neuronal apoptosis since the presence of GAPDH into the nucleus is involved in the initiation of one or more apoptotic cascades [40]. There are various case studies demonstrating the role of GAPDH in several neuronal diseases as HD and PD, and an attractive hypothesis is that GAPDH binds to the mutated proteins associated with these diseases resulting in a translocation to the nucleus, where the presence of GAPDH participates in the initiation of apoptosis. Accordingly, an increase in nuclear GAPDH in postmortem PD brain associated with the degeneration-sensitive substantia nigra dopaminergic neurons has been reported [41]. Moreover, GAPDH is recognized as a major component of amyloid plaques in Alzheimer’s diseased brains and it has been also reported to interact with neurodegenerative disease-associated proteins including the amyloid-β protein precursor (AβPP). Non-native GAPDH isoforms were able to bind to soluble Aβ species, indicating a direct involvement of GAPDH in amyloid aggregation [42].

### 3.2. Non-Nuclear Roles of GAPDH

#### 3.2.1. Cytosolic GAPDH

In physiological conditions, GAPDH is a homotetrameric protein predominantly present in the cytoplasm, converting glyceraldehyde 3-phosphate (GAP) and inorganic phosphate into 1,3-bisphosphoglycerate (1,3-BPG) in the presence of NAD^+^. However, during cellular oxidative stress, GAPDH glycolytic activity can be inhibited by *S*-thiolation, a process by which protein SH groups form mixed disulfides with low-molecular-mass thiols such as glutathione, resulting in a slower glycolytic rate in favor of respiratory cellular metabolism. The inhibition of GAPDH by *S*-thiolation is readily reversible once the oxidant is removed, suggesting GAPDH as a key enzyme also acting as a sensor of the redox status of the cell [16]. Cytosolic GADPH is generally over-expressed in highly glycolytic cancer cells [43,44]. Indeed, tumor cells prefer to produce energy through glycolysis even in aerobic conditions (the Warburg effect). This is allowed by an enhanced uptake of glucose and augmented GAPDH activity, determining also the production of glycolytic intermediates, which stimulate anabolism and abnormal cancer cell proliferation. Thus, inhibiting GAPDH activity can be considered a therapeutic opportunity for the blockage of cancer cell metabolism counteracting the high glycolytic flux of cancer cells [45]. In this view, GAPDH inhibition with koningic acid (KA) showed a selective anti-tumoral outcome, unaffecting healthy cells in which glycolysis reduction did not exceed a tolerable level [46].

Cytosolic GAPDH is also involved in apoptosis in a way mainly regulated by post-translational modifications and protein–protein interaction. Indeed, GAPDH is phosphorylated by Akt2 at Thr237 in the proximity of the binding site of Siah1, preventing its bond with Siah1 and apoptosis. The formation of the complex GAPDH/Akt2 is a mechanism identified in ovarian cancer cells to favor tumor cell survival and to avoid apoptosis [47]. Another way through which cytosolic GAPDH is involved in tumor survival is the escape from caspase-independent cell death (CICD). By stabilizing Akt to its activated and phosphorylized form, overexpressed GAPDH prevents FoxO nuclear internalization regulating Bcl-6, a Bcl-xL inhibitor with anti-apoptotic functions [48].

Furthermore, many studies have demonstrated a functional link between cytosolic GAPDH and microtubules dynamics, vesicular trafficking and membrane recruitment and fusion. GAPDH can interact with tubulin and actin in normal conditions and with stress fibers during stress, which regulate its glycolytic function promoting its inactivation [49,50]. These roles in cellular trafficking are regulated by post-translational phosphorylation of the enzyme, allowing it to take part in early secretory pathway transport. Serine/threonine kinases, facilitated by Rab2, act as regulators of GAPDH-mediated secretory activity, driving the direction of membrane transport [23,50].

Recently, another specific cytosolic activity of GAPDH has been discovered: its role as chaperone with the cellular labile heme. GAPDH helps in the transport and delivery of significant pool of cytosolic heme. It binds exogenous and endogenous heme, making it available to downstream protein targets that can be cytosolic (e.g., iNOS) or nuclear. In this way, GAPDH not only protects cells from heme toxicity but also involved in its mobilization [51]. As claimed by Tristan et al., in the cytosol, GAPDH seems to have a role of homeostasis sensor capable of sending signals to different organelles based on the stresses to which the cell is subjected [16].

#### 3.2.2. Mitochondrial GAPDH

In basal conditions, the level of GAPDH in mitochondria is very low and it strongly increases during stress conditions, such as serum deprivation and DNA damage. When GAPDH is expressed endogenously, mitochondrial GAPDH induces pro-apoptotic mitochondrial membrane permeabilization (MMP) via association with voltage dependent anion channel 1 (VDAC1) [16]. Exogenous expression of mitochondria also causes loss of the inner transmembrane potential, matrix swelling, permeabilization of the inner-mitochondrial membrane, and the release of two pro-apoptotic proteins such as cytochrome c and apoptosis-inducing factor (AIF) [22]. Kohr et al. proposed the hypothesis that SNO-GAPDH interacts with mitochondrial proteins as a trans-*S*-nitrosylase, thereby conferring the transfer of SNO from the cytosol to the mitochondria increasing mitochondrial SNO levels. The authors found that the overexpression of GAPDH increases SNO for different mitochondrial proteins, including HSP60, VDAC1, and acetyl-CoA acetyltransferase, thus supporting the role of GAPDH as a potential mitochondrial trans-*S*-nitrosylase [52]. Furthermore, during cardiac ischemia and reperfusion (I/R), GAPDH is found to be significantly associated with mitochondria, promoting direct uptake of damaged mitochondria into multi-organelle lysosomal-like (LL) structures for elimination, independently of the macroautophagy pathway [53].

#### 3.2.3. Extracellular GAPDH

GAPDH can also be secreted in the extracellular microenvironment and it is also detected in the serum and in other body fluids. Secreted GAPDH exhibits different functions; one of these is related to its localization on the cellular surface of macrophages, acting as a receptor for transferrin and lactoferrin. Lactoferrin is a key protein involved in innate immune response against microbial infection. Furthermore, during inflammation, it has been discovered that GAPDH is directly implicated in the trafficking of iron-binding lactoferrin inside the cells acting as a soluble carrier [54]. In microglial cells, which are resident macrophages in brain, secretion of GAPDH is demonstrated to be induced by extracellular ATP. In particular, ATP actives its channel P2X7R that induces the release of GAPDH from activated MG6 together with the efflux of K^+^ that leads to the formation of an inflammasome. GAPDH released by microglial cells seems to be involved in the innate immune response to neuroinflammation. Curiously, in *Streptococcus pyogenes*, GAPDH can localize on cell surface and have a role in virulence, contributing to the pathogen mechanism against the host. GAPDH is able to damage host tissues modifying the cytoskeleton to affect host immune system, inhibiting the phagocytosis and to bypass the host immune surveillance [55]. All these properties suggest a role for GAPDH as a mammalian immune system modulator.

#### 3.2.4. Other GAPDH Cellular Localizations

GAPDH have been also found to interact with tubulin and actin favoring microtubule bundling and actin polymerization [56,57,58]. Indeed, GAPDH interacts with microtubule helping in the direct management of glycolysis and helping in maintaining the quaternary structure of GAPDH by promoting the reversible dissociation of its tetrameric isoform into glycolytically inactive monomeric molecules of GAPDH. Tyrosine phosphorylation of GAPDH by atypical protein kinase Cι (aPKC) is facilitated by Rab 2, which increases phospho-GAPDH recruitment to VTCs. This process plays an important role for membrane trafficking between the ER and Golgi complex and for membrane trafficking from VTCs [16].

## 4. The Double Role of Autophagy in Cancer

Autophagy is a highly conserved catabolic mechanism that involves the formation of double-membrane vesicles, the autophagosomes, by which cellular materials are delivered to lysosomes for degradation and recycled into metabolic and biosynthetic pathways. Cytoplasmic materials are delivered to the lysosome through various type of autophagy: macroautophagy, microautophagy and chaperone-mediated autophagy (CMA). Among these various types of autophagy, macroautophagy (autophagy hereafter) is the most extensively analyzed and the major catabolic mechanism used by eukaryotic cells to maintain nutrient homeostasis and organellar quality control. On the contrary, CMA is not mediated by the autophagosome and the cytoplasmic substrates are recognized by chaperone proteins, such as Hsc70, and directed toward translocation into the lysosome for degradation [59].

Autophagy is mediated by a set of conserved genes. From yeast genetic studies to those on mammalian, the breakthrough in elucidation of the molecular machinery in autophagy came from the discovery of 35 autophagy-related (*ATGs*) genes. All *ATG* genes are required for the different steps of the autophagy: among them, *ATG 1–10*, *12–14*, *16–18*, *29* and *31* are essential for the efficient formation of autophagosome. The lysosomal degradation pathway is usually described as involving a set of about 16–20 core conserved genes. The ATG proteins encoded by these genes are traditionally classified into distinct biochemical and functional groups that act at specific stages of the autophagic flux [59,60]. The formation and turnover of the autophagosome is divided into five distinct stages: (i) initiation due to starvation conditions or other stress factors, during which a decrease of glucose transport results in the release of mTOR inhibition of the ULK1 complex; (ii) nucleation of the autophagosome by ULK1 and class III PI3K complexes; (iii) expansion and elongation of the autophagosome membrane mediated by two ubiquitin-like conjugation systems, the first system being represented by LC3I/PE and LC3II complex, while the second one involving ATG5-ATG12 conjugate mediated by ATG7 and ATG10 genes; (iv) closure and autophagosome fusion with lysosome to form an autophagolysosome by the SNARE protein syntaxin 17 (STX17); and (v) degradation of intravesicular products due to the low pH of the lysosome [61].

Autophagy has opposite and context-dependent roles in cancer: under certain circumstances, autophagy may be detrimental either via its prosurvival effects or via possible cell-death promoting effects. Thus, the role of autophagy in cancer is complex and controversial. Autophagy was originally thought to represent only a tumor suppression mechanism since Aita et al. and Liang et al. found an allelic loss of an autophagic gene, *BECN1* (*ATG6*), whose position was in close proximity to the tumor suppressor breast cancer 1 gene (*BRCA1*) [8,62].

In the early stages of neoplastic transformation, autophagy can act as a mechanism to counteract tumorigenesis by preventing the accumulation of damaged proteins and organelles and the excessive production of ROS that can promote DNA mutations and thus the development of neoplastic cells. In this way, autophagy limits oncogenic signaling and suppresses the onset of cancer. This may suggest a role for the stimulation of the autophagic process in the prevention of cancer occurrence [63]. Over the past 10 years, significant progress has been made in understanding the molecular mechanisms of autophagy and one conceptual advance is that autophagy can act also as a tumor promotion mechanism. The ability of autophagy to support cell survival under unfavorable environmental conditions, such as lack of nutrients or oxygen, which are extremely frequent in a growing tumor could help the survival of cancer cells. The tumors therefore exploit autophagy to their own advantage to promote their survival through the self-production of metabolic substrates necessary for the sustenance and spread of the tumor. Although it has been recognized that autophagy has an impact on the regulation of cell growth, the precise role of autophagy is highly contextual. Indeed, the aberrant stimulation of autophagy can determine an excessive auto-degradative event and self-eating mechanism supporting cell death.

Knocking down the expression of essential autophagy genes or deleting them can reduce tumorigenesis, confirming the functional importance of autophagy in tumor promotion. Autophagy is also upregulated in hypoxic tumor regions where it is required for tumor cell survival [64]. Thus, both the activation of cancer pathways within tumor cells and stress in the tumor microenvironment can increase the requirement for autophagy to promote tumor growth and survival [63].

Our group demonstrated that the inhibition of expression of autophagic genes by mutant p53 increases the proliferation of pancreatic cancer cells [14]. These results support the hypothesis of a new mechanism by which oncogenic mutant p53 protein promotes tumor proliferation with the concomitant inhibition of autophagy. The discovery of this double role of autophagy in human cancers has already led to the development of promising new cancer drugs so far. In another recent study, Ranieri et al. identified a new biological mechanism, which acts on the blocking of autophagic protective process and consequently inducing cell death and the reduction and elimination of the tumor [65]. In this study, they also identified a new pharmacologically active molecule, which can modulate this process resulting in a specific antitumor activity in melanoma cells.

## 5. GAPDH-Mediated Autophagy

The moonlight GAPDH protein is one of the regulators of autophagy. Besides playing the glycolytic role in the cytosol, GAPDH participates in several non-glycolytic functions including autophagy [66]. Various conditions exhibit a correlation between autophagy and the translocation of GAPDH in different subcellular compartments, especially to nucleus. For instance, GAPDH negatively regulates autophagy through the interaction with the key autophagy component ATG3 in plants [67,68]. Indeed, ROS affects the interaction between cytoplasmic GAPDH and ATG3, making free ATG3 proteins available for use in autophagy. In cardiomyocytes, oxidative stress conditions induce mitochondrial association with GAPDH promoting mitophagy, the selective degradation of mitochondria by autophagy. The formation of multiorganellar lysosomal-like (LL) structures for elimination of damaged mitochondria, also by GAPDH, helps cardiomyocytes to survive during reperfusion or reoxygenation-induced injury [53]. In brain, autophagic cytotoxic effect of cocaine is mediated by the nitric oxide-GAPDH signaling pathway [69]. Several molecular mechanisms may trigger or prevent GAPDH nuclear translocation to regulate autophagic events particularly in cancer. For instance, we discovered that the oncogenic mutant p53 protein prevents GAPDH-mediated autophagy in pancreatic cancer cells blocking the nuclear translocation of GAPDH through the stimulation of AKT and inhibition of AMPK signaling pathways [39,70], which are reported to directly phosphorylate GAPDH resulting in the inhibition or in the stimulation of GAPDH nuclear translocation, respectively [47,71]. Furthermore, in the same cancer type the production of ROS by inhibition of the antioxidant mitochondrial uncoupling protein 2 (UCP2) stimulates the nuclear translocation of GAPDH which promotes autophagy [38].

The molecular mechanisms involved in GAPDH-mediated autophagy have not yet been clarified. However, GAPDH is supposed to regulate autophagy through the direct interaction of regulatory proteins in the nucleus. In chaperone-mediated autophagy (CMA), oxidized GAPDH may be a specific substrate of this proteolytic pathway. This occurs in various steps, which include binding of the enzyme to the lysosomal membrane through the interaction with monomeric LAMP-2A (lysosome-associated membrane protein type 2A), uptake into lysosomal matrix, and degradation inside this cellular compartment [72,73]. Both heat shock cognate proteins, hsc70 and hsp90, play critical roles in the dynamics of these steps.

In low-glucose conditions, GAPDH stimulates autophagy by different pathways. One is the inhibition of mTOR signaling by the interaction between GAPDH and the Ras superfamily of GTPases Rheb, preventing Rheb binding to mTOR [74] and regulating the cross talk between glycolysis pathway and the mTORC1 pathway. In this way, GAPDH may stimulate autophagy, as mTOR inhibition causes autophagic induction [75]. In addition, under glucose deficiency GAPDH is phosphorylated by AMPK and it translocates into the nucleus, binds and activates SIRT1 deacetylase, disassociating its inhibitor DBC1 [29]. Consequently, activated SIRT1 is known to stimulate autophagy, i.e., through the deacetylation of the key autophagy component LC3 in the nucleus, which is essential for its redistribution to the cytoplasm and association with autophagic membranes [76,77]. Thus, GAPDH-mediated SIRT1 activation favors the initiation of autophagy. Interestingly, since autophagy might have a role in the promotion of cancer cell survival [4], GAPDH may act as a prosurvival factor in cancer through the induction of autophagy to support the energy consumption by rapid cell proliferation. Colell et al. showed that nuclear GAPDH protects cells from caspase-independent cell death (CICD), inducing autophagy [78]. Specifically, the nuclear function of GAPDH in protecting cells from CICD is mediated by the transcriptional up-regulation of Atg12. Since nuclear GAPDH has been involved in transcriptional regulation [28], the authors suggested that GAPDH may transcriptionally regulate ATG12 directly or indirectly. Thus, GAPDH coordinates two metabolic pathways producing ATP by glycolysis and removing damaged mitochondria by autophagy to shift the cells away from CICD [78,79]. Furthermore, Bertin et al. showed that in colon carcinoma cells the increase of GAPDH expression is sufficient to induce autophagy in vitro and in vivo [80]. Even in esophageal cancer tissues, there is a strong correlation between overexpression of GAPDH and upregulation of autophagy-related genes, like ATG12 and PIK3C3 (phosphatidylinositol 3-kinase catalytic subunit type 3) [81]. Thus, the knock-down of GAPDH was found to be sufficient to reduce autophagy and ATP levels in tumor cells [82]. Indeed, the usage of koningic acid (KA), a specific GAPDH inhibitor, inhibited autophagic flux in neuroblastoma cells [83].

**Figure 2:**
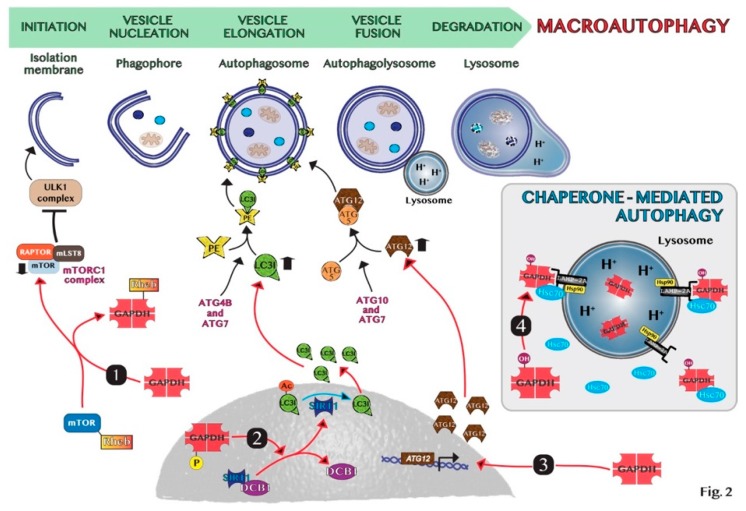
Schematic representation of molecular mechanisms of GAPDH-mediated autophagy: (1) GAPDH binds to Rheb, preventing Rheb binding to mTOR. (2) Phosphorylated GAPDH by AMPK translocates into the nucleus and disruptions the link between SIRT1 and DBC1. SIRT1 deacetylates LC3, which sustains autophagosome formation. (3) GAPDH enters the nucleus, favoring the induction of ATG12 gene. (4) In chaperone-mediated autophagy (CMA), GAPDH binds to LAMP-2A receptor on the lysosomal membrane. The chaperon proteins hsc70 and hsp90 are essential for CMA activity. Cytosolic Hsc70 after binding to CMA-substrates, such as GAPDH, transfers the complex to LAMP-2A. Hsp90 stabilizes LAMP-2A at the luminal side of the lysosomal membrane for the translocation of substrates.

In summary, the manipulation of autophagy through the regulation of the nuclear translocation of GAPDH may be an important therapeutic application in oncology. For instance, we discovered that the combined treatment with target drugs, i.e., genipin and everolimus, which are inhibitors of UCP2 and mTOR, respectively, promotes GAPDH nuclear translocation stimulating the formation of autophagic vesicles and pancreas cancer cell death [38]. On the contrary, when tumors promote autophagy as a survival mechanism, the modulation of nuclear GAPDH may be a useful target to counteract autophagy. Indeed, Guan et al. showed that GAPDH-siRNA encapsulated in nano-targeted liposomes reduces the autophagic flux in cancer cells and intriguingly favors the outcome of cancer cell drug resistance [82]. Thus, GAPDH may constitute a valuable target to modulate autophagy in cancer therapy and the main mechanisms at the basis of GAPDH-mediated autophagy regulation are schematically shown in Figure 2.

## 6. Aggregation Mechanisms of GAPDH and Impact on Diseases

**Figure 3:**
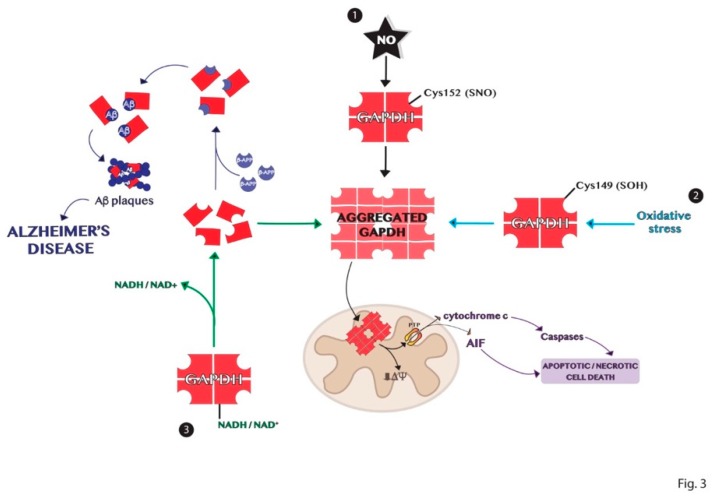
Schematic representation of the mechanisms of GAPDH aggregation. (1) Nitrosylation of Cys152 in the active site of GAPDH is crucial for its aggregation induced by nitric oxide. (2) Oxidation of the redox-sensitive Cys149 of GAPDH induced by oxidative stress is involved in its aggregation. (3) GAPDH without its cofactor NADH or NAD+ is susceptible to denaturation and consequent aggregation. Dimer or monomers of GAPDH can aggregate or bind other biomolecules in neurodegenerative diseases, as Alzheimer’s disease.

Protein aggregation is another process of the multiple functions involving GAPDH [84]. The high-resolution structure of the enzyme shows a highly stable tetrameric protein with identical subunits, each constituting an active site [40]. Tetrameric dissociation of the enzyme produces dimers and monomers that aggregate or bind to other biomolecules such as proteins and nucleic acids [16,85]. The apoform of the enzyme without its cofactors NAD^+^ or NADH, is susceptible to denaturation and consequently aggregation. These events are dependent on the content of cofactors and other ligands and on the presence of different signal molecules such as ROS that may influence the amyloidogenic processes through intermolecular disulfide bonds [25,84]. Indeed, GAPDH is an intracellular sensor of oxidative stress as discussed above. Some cysteine residues of the active site of the enzyme are strongly susceptible to various types of oxidation, decreasing the enzyme affinity to the cofactor and promoting its dissociation. The active site Cys152 of GAPDH is crucial for its aggregation induced by nitric oxide [86]. Furthermore, the site-directed mutagenesis experiments revealed that Cys149 is one of the most aggregate-prone cysteine residue of GAPDH induced by oxidative stress [25]. Several pieces of evidence show the involvement of GAPDH in the development of neurodegenerative disorders such as Alzheimer’s, Huntington’s, and Parkinson’s diseases, which are characterized by the accumulation of protein aggregates [87,88,89]. Indeed, denatured GAPDH forms may bind soluble Aβ species yielding insoluble aggregates [90,91]. The deposition of β-amyloid proteins is one of the most distinct features in Alzheimer’s disease and results from proteolytic processing of β-amyloid precursor protein (β-APP) [91]. GAPDH binds β-APP altering the normal processing of β-APP to produce β-amyloid protein and this interaction was found in amyloid plaques from the brains of patients with AD [42,92,93]. Furthermore, GAPDH aggregates promote the formation of Lewy bodies in the brains of individuals with PD [88]. GAPDH–protein interactions also occur with spinocerebellar ataxia type-1 and spinobulbar muscular atrophy gene products [94,95,96]. In conclusion, the comprehension of the mechanisms involved in the formation of GAPDH aggregates, represented in Figure 3, may help in the understanding of the biological alterations observed in neurodegenerative diseases.

### GAPDH Aggregates and GAPDH-Mediated Cell Death in Neurodegenerative Diseases

The amyloid-like GAPDH aggregates enhance amyloidogenesis and promote mitochondrial dysfunction and cell death [97]. The detailed mechanisms are still unknown. Nakajima et al. showed that GAPDH-overexpressing HeLa cells induce the formation of GAPDH aggregates in both nucleus and cytoplasm under oxidative stress conditions and the degree of GAPDH aggregation is correlated with that of the oxidative stress-induced cell death [25]. In particular, oxidized GAPDH may translocate to the nucleus and bind Siah1 inducing cell death mechanism independent of glycolytic deficit [35]. In HD, overexpression of GAPDH or Siah1 enhances huntingtin (Htt) nuclear translocation and cytotoxicity [89]. In fact, Htt and other proteins with polyglutamine repeats are reported to bind to GAPDH [95]. The authors showed that the binding of GAPDH to an N-terminal fragment of Htt was relevant to the nuclear targeting of Htt and its cytotoxicity. Remarkably, neuroprotective actions of several anti-apoptotic drugs involve the blockade of the GAPDH/Siah1 system and inhibit GAPDH aggregation to reduce the effects of neurodegenerative diseases [98,99]. The NO-induced aggregates of GAPDH mainly localize to the mitochondria and induce a decrease in mitochondrial membrane potential (Δψ) and mitochondrial swelling through the opening of mitochondrial permeability transition pore (mPTP) with subsequent cytosolic release of cytochrome c and nuclear translocation of apoptosis-inducing factor (AIF) and causing apoptotic/necrotic cell death [98]. Furthermore, unlike aggregates of amyloidogenic proteins [100,101], the authors discovered that GAPDH aggregates do not influence NO stress-induced proteasome activity, ER stress-related protein expression or the induction of autophagy [102]. Hwang et al. discovered that in HD, the expanded polyglutamine repeats associate with oxidized and inactive mitochondrial GAPDH, blocking GAPDH-induced mitophagy, leading to accumulation of damaged mitochondria and increasing cell death. The authors further demonstrated that overexpression of inactive GAPDH rescues this process and enhances mitochondrial function and cell survival, indicating a role for GAPDH-driven mitophagy in the pathology of HD [103].

## 7. Conclusions and Future Perspectives

Numerous studies highlight the different functions and key roles of GAPDH in many molecular processes across all the compartments of the cell [16]. Indeed, the functional versatility of this enzyme is mainly related to its translocation to different subcellular compartments and has implications in several diseases such as cancer or neurodegenerative disorders. In this article, we describe the mechanisms and the pathological implications of GAPDH-mediated autophagy and GAPDH aggregation, which may influence cancer cell growth and neurodegenerative disorders. Cancer-related factors can modulate GAPDH nuclear translocation, which is fundamental to regulate autophagy and cell death mechanisms [44]. Autophagy stimulation by nuclear GADPH may influence cancer cell fate acting as a prosurvival factor in cancer cells, supporting the energy consumption given by rapid cell proliferation even in stressing conditions. Besides cancer, GAPDH nuclear translocation is a critical event in neurodegenerative diseases. Specifically, augmented nuclear GAPDH is associated with neuronal cell death [104]. Moreover, the formation of aggregates of GAPDH or the interaction of GAPDH with specific disease-related proteins is involved in neuronal cell death and mitochondrial dysfunction [97,105], causing the development of different neurodegenerative diseases. Intriguingly, from a therapeutic point of view, GAPDH can be considered as a potential therapeutic target to work on multiple issues and in several diseases. Finally, the knowledge of GAPDH versatility is a necessary step to design compounds and to modulate its pleiotropic functions in diseases.
